# The Maize *glossy13* Gene, Cloned via BSR-Seq and Seq-Walking Encodes a Putative ABC Transporter Required for the Normal Accumulation of Epicuticular Waxes

**DOI:** 10.1371/journal.pone.0082333

**Published:** 2013-12-06

**Authors:** Li Li, Delin Li, Sanzhen Liu, Xiaoli Ma, Charles R. Dietrich, Heng-Cheng Hu, Gaisheng Zhang, Zhiyong Liu, Jun Zheng, Guoying Wang, Patrick S. Schnable

**Affiliations:** 1 College of Agronomy, Northwest Agriculture & Forestry University, Yangling, Shaanxi, China; 2 Department of Agronomy, Iowa State University, Ames, Iowa, United States of America; 3 Department of Plant Genetics & Breeding, China Agricultural University, Beijing, Hebei, China; 4 Institute of Crop Sciences, Chinese Academy of Agricultural Sciences, Beijing, Hebei, China; 5 Center for Plant Genomics, Iowa State University, Ames, Iowa, United States of America; China Agricultural University, China

## Abstract

Aerial plant surfaces are covered by epicuticular waxes that among other purposes serve to control water loss. Maize *glossy* mutants originally identified by their “glossy” phenotypes exhibit alterations in the accumulation of epicuticular waxes. By combining data from a BSR-Seq experiment and the newly developed Seq-Walking technology, GRMZM2G118243 was identified as a strong candidate for being the *glossy13* gene. The finding that multiple EMS-induced alleles contain premature stop codons in GRMZM2G118243, and the one knockout allele of *gl13*, validates the hypothesis that gene GRMZM2G118243 is *gl13*. Consistent with this, GRMZM2G118243 is an ortholog of AtABCG32 (*Arabidopsis thaliana*), HvABCG31 (barley) and OsABCG31 (rice), which encode ABCG subfamily transporters involved in the trans-membrane transport of various secondary metabolites. We therefore hypothesize that *gl13* is involved in the transport of epicuticular waxes onto the surfaces of seedling leaves.

## Introduction

Plant cuticles provide the first line of defense between a plant and its environment, providing protection from, for example, water loss [1-3] and UV irradiation [4]. The plant cuticle also plays critical roles in plant interactions with the biotic environment, including bacterial, fungi and insects [5]. As such, the physical and chemical properties of cuticle are of particular interest in a world that is experiencing global climate change. The cuticle consists of a thin layer of secreted compounds on the surface of the epidermis, and is synthesized exclusively by the epidermal cells [6,7]. The cuticle proper is covered by microcrystalline epicuticular waxes [7]. 

Epicuticular waxes are composed of several classes of compounds, including very-long-chain fatty acids (VLCFA; >18C), esters, primary and secondary alcohols, fatty aldehydes and ketones [8]. Different species have distinct epicuticular waxes compositions and epicuticular wax composition can also differ among tissues within the same species [9]. In *Arabidopsis* and barley (*Hordeum vulgare*), ~20 and ~80 independent *eceriferum* (*cer*) loci that affect wax accumulation have been defined genetically, respectively [9,10,11]. In maize (*Zea mays*), more than 30 *glossy* (or *gl*) loci have been identified that affect the quantity and/or the composition of eipcuticular waxes on the surfaces of seedling leaves [12], (and Schnable lab, unpublished data). 

Seven of the maize glossy genes have been cloned [13-18]. *gl1* encodes a protein that is similar to an *Arabidopsis WAX2*-related protein hydroxylase displaying transmembrane domains [4]. The *gl2* gene encodes a protein with transferase activity whose expression is restricted to epidermal cells and is involved in epidermal wax formation only in the green portions of seedlings [14]. *gl3* is a cell autonomous [12], putative *myb* transcription factor that affects the expression of other *glossy* genes [17]. *gl4* is a homolog of the *Arabidopsis CUT1* gene which encodes a condensing enzyme involved in the synthesis of very-long-chain fatty acids [18]. *gl8a* encodes a β-ketoacyl reductase of the fatty acid elongase complex involved in wax production [16]. *gl15* encodes an *APETALA2*-like transcription factor involved in the transition from juvenile to adult leaves [19]. 

As a first step in cloning the *gl13* gene a collection of EMS-induced [20] and *Mu*-induced [21] mutants was isolated. BSR-Seq was used to map the *gl13* locus to an 8 Mb interval of chromosome 3. Subsequently, a newly developed technology, Seq-Walking was used to identify *Mu* insertions within the 8 Mb mapping interval of a *Mu*-induced allele of *gl13*, thereby defining a *gl13* candidate gene. Sequence analyses of multiple EMS-induced alleles confirmed that the candidate gene is *gl13*. The *gl13* gene is a homolog of an ATP-Binding cassette G (ABCG32) family *Arabidopsis* gene, suggesting that *gl13* is involved in the transport of epicuticular waxes onto the surfaces of epidermal cells.

## Materials and Methods

### Genetic stocks

**Figure 1:**
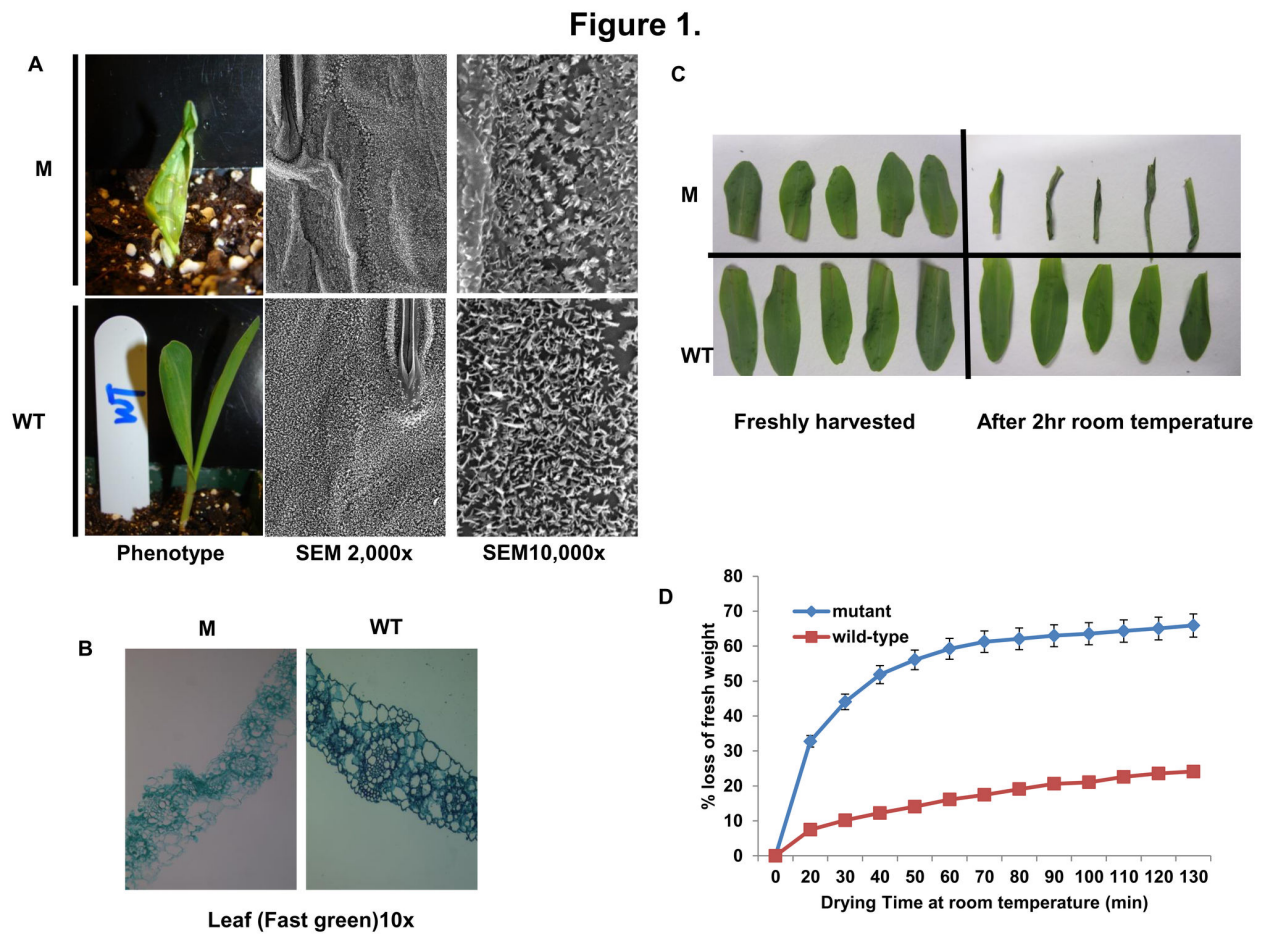
BSR-Seq Mapping and Seq-Walking of *gl13*. (A) Numbers of informative SNPs used in BSR-Seq analyses of 3 EMS-induced *gl13* alleles. (B) Results of BSR-Seq analysis for three alleles of *gl13*. (C) Crossing strategy used to generate seedlings homozygous for wild-type and *gl13-Mu 90-3134B1* alleles for Seq-Walking. (D) Identification of shared, wild-type specific, and mutant-specific Mu-flanking sequence (MFS) sites and identification of mutant-specific MFS located with the *gl13* mapping interval.

The *gl13-ref* allele (AC520*/gl13-N169*; Maize Genetics Stock Center ID U440B; Schnable Lab #Ac *1200-1205*) was induced by EMS from an uncertain of progenitor by Gerry Neuffer. A single *Mu*-induced allele of *gl13* (*gl13-Mu 90-3134B1, ID# 91g-6608*) was isolated from the Q60 genetic background by the Schnable lab in 1990. A plant heterozygous for this allele was crossed to Q60 and a resulting heterozygous progeny was self-pollinated to create an F2 population. Wild-type progeny (*Gl13*/*Gl13*) from non-segregating F3 families and mutant plants (*gl13-Mu/gl13-Mu*) from a segregating F3 family were used for Seq-Walking (Figure 1C). Eight EMS-induced alleles (*gl13-94-1001-1481*, *gl13-2:207-44, gl13-N211B, gl13-2:15-33, gl13-2:207-44, gl13-2:225-43, gl13-N1478B* and *gl13-N169* or *gl13-ref*) and two spontaneous *gl13* alleles (*gl13-Nec8495, gl13-U440B/PI251938b*) derived from different labs as described by Schnable et al. [12] were used in this study. 

### BSR-Seq and Differential Gene Expression Analysis

Three EMS-induced alleles (*gl13-94-1001-1481*, *gl13-2:207-44* and *gl13-N211B*) were used for BSR-Seq mapping and expression analysis. Approximately 35 mutant and 35 wild-type seedlings per segregating F2 population were grown in a greenhouse sand bench under 27°C days and 24°C nights with 15 hours of daylight. Above ground tissues were collected 8 DAP (Days After Planting) for the isolation of RNA. Total RNAs were quantified with a Bioanalyzer and then subjected to RNA-Sequencing using an Illumina HiSeq2000 paired-end sequencing instrument. Sequencing data were trimmed and aligned to the B73 reference genome (AGPv2) and subjected to SNP calling as well as reads count normalization [17]. SNPs detected between the B73 reference genome and the 25 NAM founders (plus Mo17) were used for SNP filtering. Final filtered SNPs from both mutants and wild-type pools were used for BSR-Seq analysis [17]. Genes with an average of at least five uniquely mapped read across samples were tested for differential expression between the *gl13* mutant and sibling wild-type seedlings using the R package QuasiSeq (http://cran.r-project.org/web/packages/QuasiSeq). The negative binomial QLSpline method implemented in the QuasiSeq package was used to compute a *p-value* for each gene. The 0.75 quantile of reads from each sample was used as the normalization factor [22]. A multiple test controlling approach [23] was used to convert *p-values* to *q-value*. To obtain approximate control of the false discovery rate at 5%, genes with *q*-values no larger than 0.05 were declared to be differentially expressed. Gene Ontology (GO) terms were determined using tools and resources available at: http://geneontology.org/

### Seq-Walking Strategy

To identify the causal *Mu* insertion in *gl13-Mu 90-3134B1*, homozygous F2 wild-type and mutant plants derived from same F2 family were used for Seq-Walking (Figure 1C). Genomic DNAs from mutant and wild-type pools were fragmented via sonication (BioRuptor UCD-200, Diagenode, USA) for 8-10 cycles at intervals of 30s /30s on/stop. Purified DNA fragments were used for library preparation using the NEBNext® DNA Library Prep Master Mix Set for Illumina® kit (NEW ENGLAND BioLabs® inc.). The Seq-Walking library was prepared according to Ion Torrent library preparation pipeline (Figure S1). Equal quantities of DNA (based on results from the Bioanalyzer’s 2100 expert High Sensitive DNA Assay) from the mutant and wild-type pools (each of which had distinct DNA barcodes) were pooled. Final libraries were sequenced on an Ion Torrent PGM instrument using a 318 Chip. 

### Data analysis

Raw PGM reads were decoded based on library-specific barcodes with an in-house Perl script. After trimming barcodes, *Mu* sequences from the decoded reads were trimmed using the software package, SeqClean (sourceforge.net/projects/seqclean). Those reads that were longer than 90 bp after trimming were aligned to the B73 reference genome (AGPv2) using the aligner GSNAP [24]. Uniquely mapped reads were used to define a set of non-redundant Mu-flanking sequence (MFS) sites for each library. The read count for each MFS site was determined for each library. 

### Sanger sequencing

DNA and total RNA samples isolated from the 10 *gl13* alleles that were not derived from *Mu* stocks were used as templates for PCR amplification with a set of gene-specific primers that cover the transcribed region of the *gl13* gene (Table S11). 

An MFS site from the *Mu*-insertion allele, *gl13-Mu 90-3134B1* was amplified using a *Mu* primer (*Mu*TIR: 5’AGAGAAGCCAACGCCA(AT)CGCCTC(CT)ATTTCGTC3’) [25] and gene-specific primer (gl13C1L11: 5’GGAGCAGATTCTTGGAGTGG3’ or gl13C1R11: 5’CCGAACTGAGAGGTCAGGAG3’ (also see Table S11). The resulting PCR products were sequenced using Sanger technology. 

### Quantitative RT-PCR analysis for expression pattern in 11 *gl13* alleles

Seedlings from an F2 family segregating for *gl13-ref* were grown in a growth chamber under 18 hours of light at 35°C and 6 hours of dark at 20°C and then harvested at 6 DAP, 10 DAP and 15 DAP. Similarly, 11 F2 families each of which was segregating for one of the 11 alleles (Table 1) were grown in a growth chamber under 18 hours of light at 35°C and 6 hours of dark at 20°C. At 7~8 DAP seedlings were screened for phenotype and mutant and wild-type seedlings were harvested at 10 DAP. Total RNA was isolated from tissue samples using the RNeasy Mini Kit (Cat.No.74134, Qiagen, USA). cDNA was prepared using the iScript cDNA Synthesis Kit (Bio-Rad). Real-Time PCR was performed using iQ SYBR Green Supermix (Bio-Rad) on the Light Cycler® 480II instrument (Roche, USA). Relative quantification values (RQ) were calculated by the 2^−ΔCt^ method (RQ = 2^−ΔCt^) with two biological replicates and two technical replicates using actin mRNA as an endogenous control. Oligonucleotides used for real-time PCR: forward primer: gl13C1L13.2, 5’ACCATTGCGCCTATTATTGC3’; reverse primer: gl13C1R11, 5’CCGAACTGAGAGGTCAGGAG3’ (also in Table S11); Actin408F: 5’CCAGGCTGTTCTTTCGTTGT3’; Actin520R: 5’GCAGTCTCCAGCTCCTGTTC3’

**Table 1:** Sequence Characterization of 11 *gl13* mutant alleles.

**ID**	**Allele**	**Progenitor **	**Mutagen **	**Pos. (bp) On Chr3 **	**Mutation**	**Ref. (B73)**	**A632**	**Mo17**	**Lesion**	**AA change**
**1**	*gl13-94-1001-746* ^a^	*A632*	**EMS**	10282947-10282948	del^d^	AC	AC	AC	2 bp deletion in 47 bp downstream of stop codon	-
**2**	*gl13-94-1001-1481* ^a^	*A632*	**EMS**	10273165	G->A	G	G	G	28 bp upstream of start codon	-
**3**	*gl13-2:15-33* ^b^	*Mo17*	**EMS**	10282710	G->A	G	G	G	Non-synonymous	1369AA(G/S)
**4**	*gl13-2:207-44* ^b^	*Mo17*	**EMS**	10277900	G->A	G	G	G	PTC	529 AA
**5**	*gl13-2:225-43* ^b^	*Mo17*	**EMS**	10278657	C->T	C	C	C	PTC	655AA(Q/PTC)
**6**	*gl13-N211B* ^a^	ND^e^	**EMS**	10276510	C->T	C	C	C	PTC	374AA(Q/PTC)
**7**	*gl13-N1478B* ^a^	ND	**EMS**	10279970	C->T	C	C	C	PTC	985AA(Q/PTC)
**8**	*gl13-Nec 8495* ^a^	ND	**ND**	10282712	C->A	C	C	C	synonymous	NO CHANGE
**9**	*gl13-U440B* ^c^	ND	**ND**	10282712	C->A	C	C	C	synonymous	NO CHANGE
**10**	*gl13-N169 (gl13-ref*)^a^	ND	**EMS**	10281826	G->A	G	G	G	Non-synonymous	1335AA(G/R)
**11**	*gl13-Mu 90-3134B1* ^c^	*Q60*	***Mu***	10281062-10281070	-	-	-	-	*Mu* insertion (*Mu1*)	-

^a^Obtained from Dr. Gerry Neuffer; ^b^Obtained from Dr. Allen Wright; ^c^Generated by the Schnable lab. ^d^AC deletion (47 bp downstream of stop codon), was also detected in 10/25 of the NAM parents, but not in its progenitor A632; ^e^ND, unknown.

### Paraffin sectioning

Fresh tissues from leaf blades and stems of 10 DAP seedlings were subject to FAA fixation followed by dehydration with gradient alcohol solutions (50%, 70%, 95%, 100%), and then infiltrated with 50% xylene solution (1:1 of xylene:alcohol) and subsequently 100% Xylene. Tissues were embedded in a mixture of paraffin and xylene at 40°C overnight, followed with 60°C paraplast infiltration using pure paraffin. Imbedded tissues were sectioned on a Leica RM2135 rotary microtome (Thermo Scientific, USA), stained with Fast Green and imaged on Nikon Eclipse E1300 Microscope (Nikon, USA).

## Results

### Isolation of *glossy13* mutants

Allelism tests of a large collection of *glossy* mutants identified 11 alleles of *gl13* (Table 1). One of these alleles (*gl13-Mu 90-3134B1*) was isolated via random mutagenesis using the *Mu* transposon [12]. Eight of these alleles were generated via EMS-mutagenesis. The remaining 2 alleles were obtained from other sources or isolated by the Schnable lab [12]. 

### Phenotypic characterization of *glossy13* mutants

**Figure 2:**
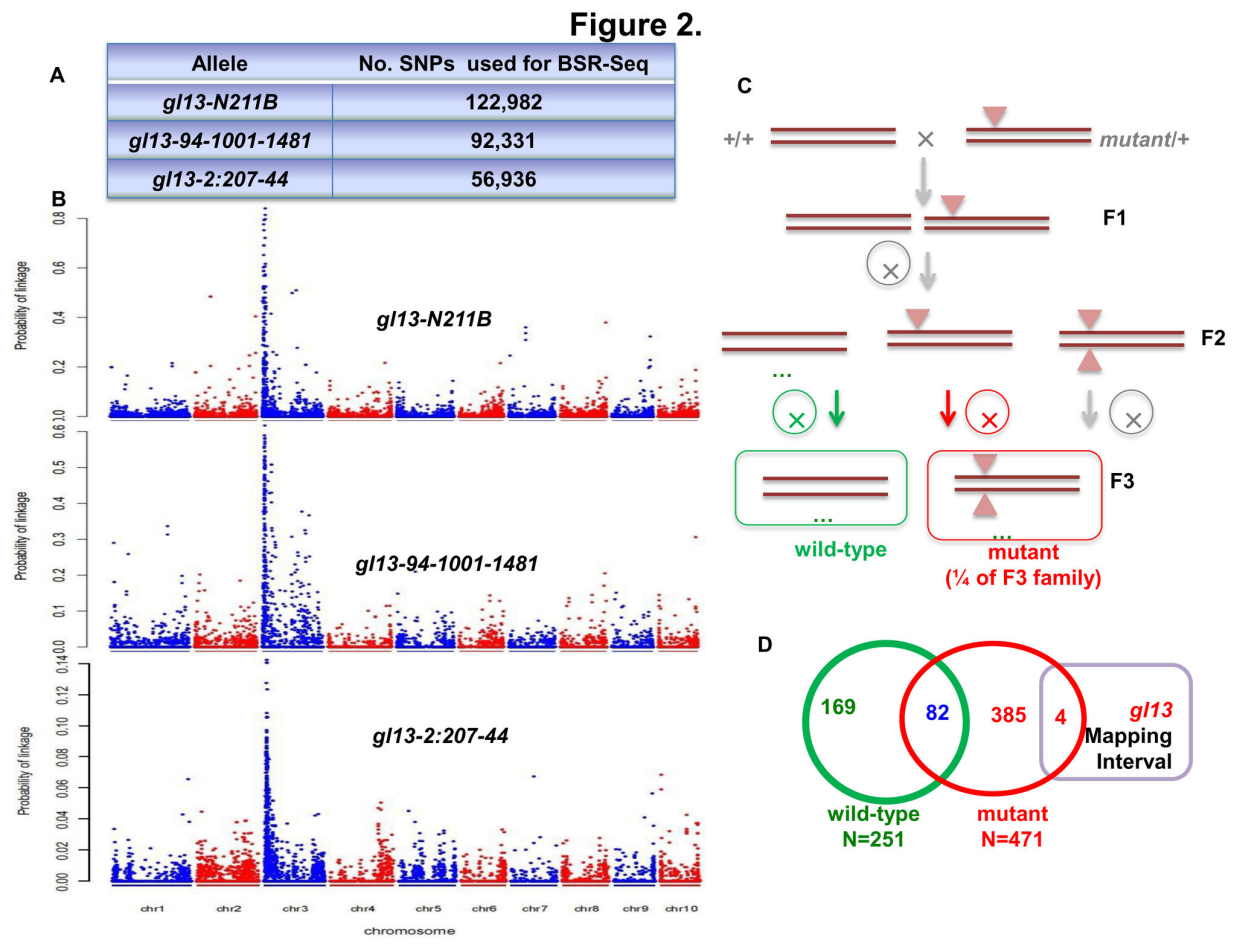
Phenotypic characterization of the *gl13-ref* mutant phenotype. (A) Comparisons of gross morphology and epicuticular wax accumulation and morphology in mutant (upper) and wild-type (lower) seedlings; (B) Comparisons of paraffin sections (10x) of leaves from mutant (left) and wild-type (right) seedlings stained with Fast Green; (C) Detached leaves from mutant (upper) and wild-type (lower) immediately after harvest and after 2 hours at room temperature; (D) Water loss from detached mutant and wild-type seedling leaves at room temperature. Error bars = SE.

Ten day-old seedlings homozygous for the *glossy13* reference allele (*gl13-N169*) exhibit a glossy phenotype (Figure 2A). Somewhat later in development, these mutant seedlings develop rolled leaves that exhibit necrotic lesions and homozygotes for some mutant alleles are ultimately lethal. Scanning electron microscopic analysis of juvenile leaves from seedlings homozygous for the *gl13-ref* allele revealed significant disruption of the epicuticular wax crystals as compared to wild-type. Paraffin sections of seedling leaves reveal significant differences in cell morphology between sibling wild-type and *gl13-ref* mutant leaves; as compared to wild-type leaves epidermal cells on mutant leaves are unordered (Figure 2B). Because epicuticular waxes serve as a water barrier, we tested the ability of the *gl13* mutant to retain water post-harvest. Detached leaf sections were exposed to the open air at room temperature for two hours and then weighed. Leaf sections from the *gl13-N169* (*gl13-ref*) mutant showed more water loss than sibling wild-type controls (Figure 2C, D). 

### Mapping *gl13* to an 8 Mb interval on chromosome 3 via BSR-Seq

BSR-Seq technology [17] is a modification of Bulked Segregant Analysis [26], because it is based on RNA-Seq data, BSR-Seq provides both genetic mapping data and global expression data [17]. BSR-Seq was conducted using three independent EMS-induced mutant alleles of *gl13* (Methods). Three F2 populations, each of which was segregating for a different EMS-induced allele were subjected to RNA-Seq. SNPs were identified between mutants and sibling wild-type controls via comparisons of RNA-Seq reads to the B73 reference genome. Identified SNPs were subjected to a statistical test for linkage to *gl13*, based on the numbers of RNA-Seq read counts associated with each SNP in each of the two pools (Methods, Figure 1A). The probability of linkage for each SNP from each of the three RNA-Seq experiments was plotted. Clusters of significant SNPs were detected in an 8 Mb region on the long arm of chromosome 3 (chr3:5-13 Mb) in the two F2 families segregating for *gl13-94-1001-1481* and *gl13-2:207-44*. The mapping interval detected in the F2 family segregating for *gl13-N211B* was larger, but overlapped with this 8 Mb region (Figure 1B). 

### Identification of the *gl13* gene via Seq-Walking

Here, we report a novel technology, Seq-Walking that amplifies and sequences DNA fragments flanking *Mu* transposon insertions throughout the genome. A set of F3 families derived from homozygous mutant and non-mutant individuals from an F2 population segregating for the *Mu*-induced allele, *gl13-Mu 90-3134B1* (Figure 1C; Methods) was used for Seq-Walking (Figure S1). Genomic DNA samples were extracted from F3 mutant plants (*gl13*/*gl13*) and wild-type plants from non-segregating F2 families (*Gl13/Gl13*). Pooled DNAs were used for NGS library preparation (Figure 1C) and sequenced using a Life Technologies PGM instrument (Methods).

561,749 raw reads were obtained from the PGM; 411,892 were from the *gl13* mutant library and 149,857 from the wild-type library. After filtering for length (>90 bp) 69,578 and 25,645 reads from the mutant and wild-type samples, respectively could be uniquely mapped to the B73 reference genome [27]. These reads are derived from 471 and 251 non-redundant MFS sites in the *gl13* mutant and wild-type pools, respectively (Figure 1D). Among these sites, 82 were detected in both mutant and wild-type pools and thus removed. The remaining non-redundant 389 MFS sites in the mutant pool and 169 MFS sites in the wild-type pool were defined as “mutant specific” and “wild-type specific”, respectively. Four of the 389 “mutant specific” insertion with highest read counts were located in the 8 Mb mapping interval defined by the BSR-Seq mapping experiment (Table 2). Among these four genes, only GRMZM2G118243 was differentially expressed between mutant and wild-type based on RNA-Seq read counts from the BSR-Seq experiment; the corresponding MFS sites also had the highest reads count in the Seq-Walking analysis (Table 2). Using PCR primers specific to the *Mu* transposon and GRMZM2G118243 (Methods) we detected a *Mu* insertion (*Mu1* [28]) in *gl13-Mu 90-3134B1* at the site predicted based on the results from the Seq-Walking experiment (Figure 1C, D). GRMZM2G118243 was therefore identified as a *gl13* gene candidate.

**Table 2:** Read counts of MFS obtained via Seq-Walking located within the 8 Mb *gl13* mapping interval.

Chr	Start of MFS (bp)^a^	End of MFS (bp)^a^	Read Count	Target gene	Insertion site	DEG^b^
chr3	4,952,465	4,952,473	23	Non-genic insertion	-	-
chr3	10,281,062	10,281,070	727	GRMZM2G118243^c^	Exon 21	Yes
chr3	14,929,161	14,929,169	402	GRMZM2G088443	Exon1	No
chr3	14,929,196	14,929,204	36	GRMZM2G088443	Exon1	No

^a^Relative to start of Mu insertion; ^b^DEG, differentially expressed gene (*gl13* vs. WT, log_2_FC>4, Table S9, S10); ^c^ *gl13* gene.

### Validation of the *gl13* candidate gene

**Figure 3:**
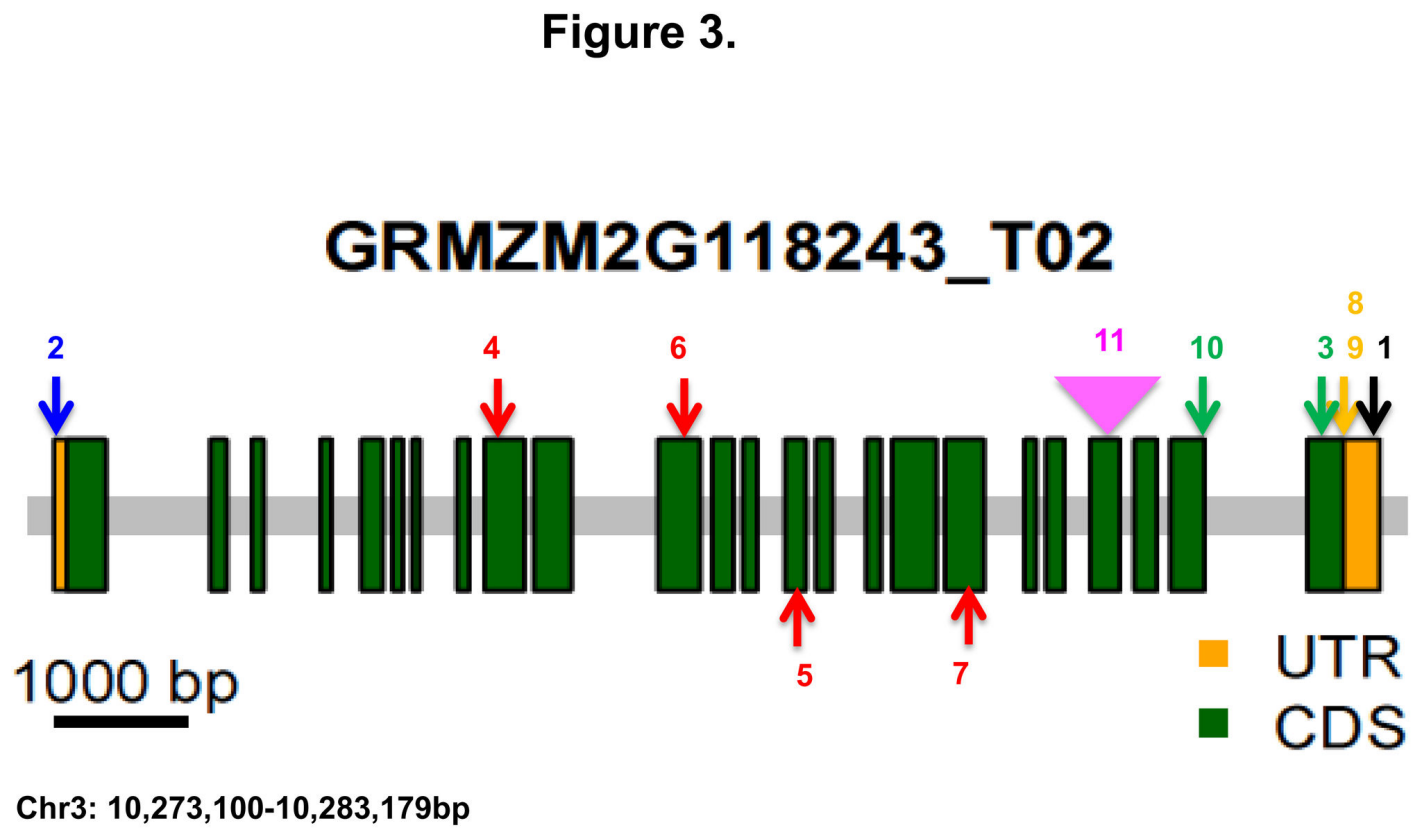
Sequence analysis of 11 *gl13* alleles. The positions of detected lesions are indicated by arrows within the *gl13* gene. Green boxes indicate gene coding region (CDS) and yellow boxes indicate un-translated region (UTR). Alleles are numbered according to Table 1. Four EMS-induced premature terminal codon (PTC) mutations are shown in red; two EMS-induced non-synonymous substitutions are shown in green; an EMS-induced G->A transition is shown in blue; the location of a *Mu* insertion (belong to Mu1 family [25]) is shown in pink; the location of a C->A transversion found in two spontaneous alleles are shown in yellow; and a 2bp deletion detected in one EMS-induced allele is shown in black.

The sequences of RNA-seq reads from the BSR-Seq mapping experiment that align to GRMZM2G118243 were compared to the progenitor alleles of the three *gl13* EMS-induced alleles used in the BSR-Seq experiment to determine whether the EMS-induced alleles contained typical EMS-induced polymorphisms (G/C -> A/T transitions [29]) relative to their progenitor alleles. The *gl13-2:207-44* and *gl13-94-1001-1481* alleles were derived from Mo17 and A632, respectively. The sequences of the Mo17 and A632 progenitors were imputed by projecting known Mo17 vs. B73 [30,31] and A632 vs. B73 (Schnable Lab, unpublished results) SNPs discovered from RNA-Seq data onto the B73 allele of GRMZM2G118243. The progenitor of *gl13-N211B* is not known. Hence, we compared the sequences of the RNA-Seq reads from this allele to the sequences of all GRMZM2G118243 alleles present in the 26 founders of the NAM population [32] that had been determined by RNA-Seq analyses (Schnable Lab, unpublished data) and excluded from consideration any SNP present in any of the NAM founders. Based on these analyses two of the three EMS-induced alleles contain typical EMS-induced mutations that result in premature termination codons (PTCs). These PTCs are located in exon 9 (*gl13-N211B*) and exon 11 (*gl13-2:207-44*) of GRMZM2G118243 (Figure 3, red arrows 4, #6, Table 1, S1). A typical EMS-induced mutation (G/C -> A/T) was detected in the 5’ *gl13-94-1001-1481* as compared with its progenitor A632 (Figure 3, 2). Because RNA-Seq data were obtained from only the transcribed region, it is also possible that a mutation outside of the transcribed region is responsible for the mutant phenotype of *gl13-94-1001-1481*.

To confirm the existence of the PTCs identified in 2/3 of the EMS-induced alleles via inspection of RNA-seq reads and discover further evidence in support of the *gl13* candidate gene, the three EMS-induced alleles used in the BSR-Seq experiment plus five additional EMS-induced alleles and two spontaneous alleles (i.e., a total of 10 alleles) were sequenced via Sanger sequencing. In addition to confirming the two PTC alleles first identified in the BSR-Seq experiment, we detected PTCs in two additional EMS-induced alleles: *gl13-2:225-43* (exon *18*), *gl13-N1478B* (exon 14) (Figure 3, red arrows 5, #7; Table 1, S1). 

G to A transitions were detected in three other alleles. *gl13-2:15-33* and the reference allele (*gl13-N169*) contain non-synonymous mutations in exons 17 and 23, respectively (Figure 3, 3, 10 Table 1, S1). The *gl13-94-1001-1481* allele, which was derived from the same genetic background as *gl13-94-1001-746*, contains a G to A transition 24 bp upstream of the start codon (Figure 3, #2; Table 1, S1).

Polymorphisms were also detected in other alleles, although it is not possible to establish whether these are causative. One EMS-induced *gl13* allele (*gl13-94-1001-746*), which was derived from A632, contains a 2 bp deletion (of AC) 47 bp downstream of the stop codon (Figure 3, #1; Table 1, S1). The two spontaneous *glossy* alleles, *gl13-Nec8495* and *gl13*-*U440B/PI251938b* both contain a synonymous substitution (A to C transversion) at the same position in exon24 (Figure 3, 8, 9; Table 1, S1). 

It is notable that even though an ~1 kb section of the *gl13* gene between exon 6 and exon 7 could not be cleanly amplified and sequenced, we were still able to identify PTC or non-synonymous mutations typical of EMS-mutagenesis in 6/8 of the EMS-induced alleles, thereby confirming that GRMZM2G118243 is *gl13*. This *gl13* gene is 11,328 bp in length (located between positions 10,272,500 to 10,283,828 bp on chromosome 3) and contains 24 exons.

### *gl13* is predicted to encode a putative G family ABC transporter

**Figure 4:**
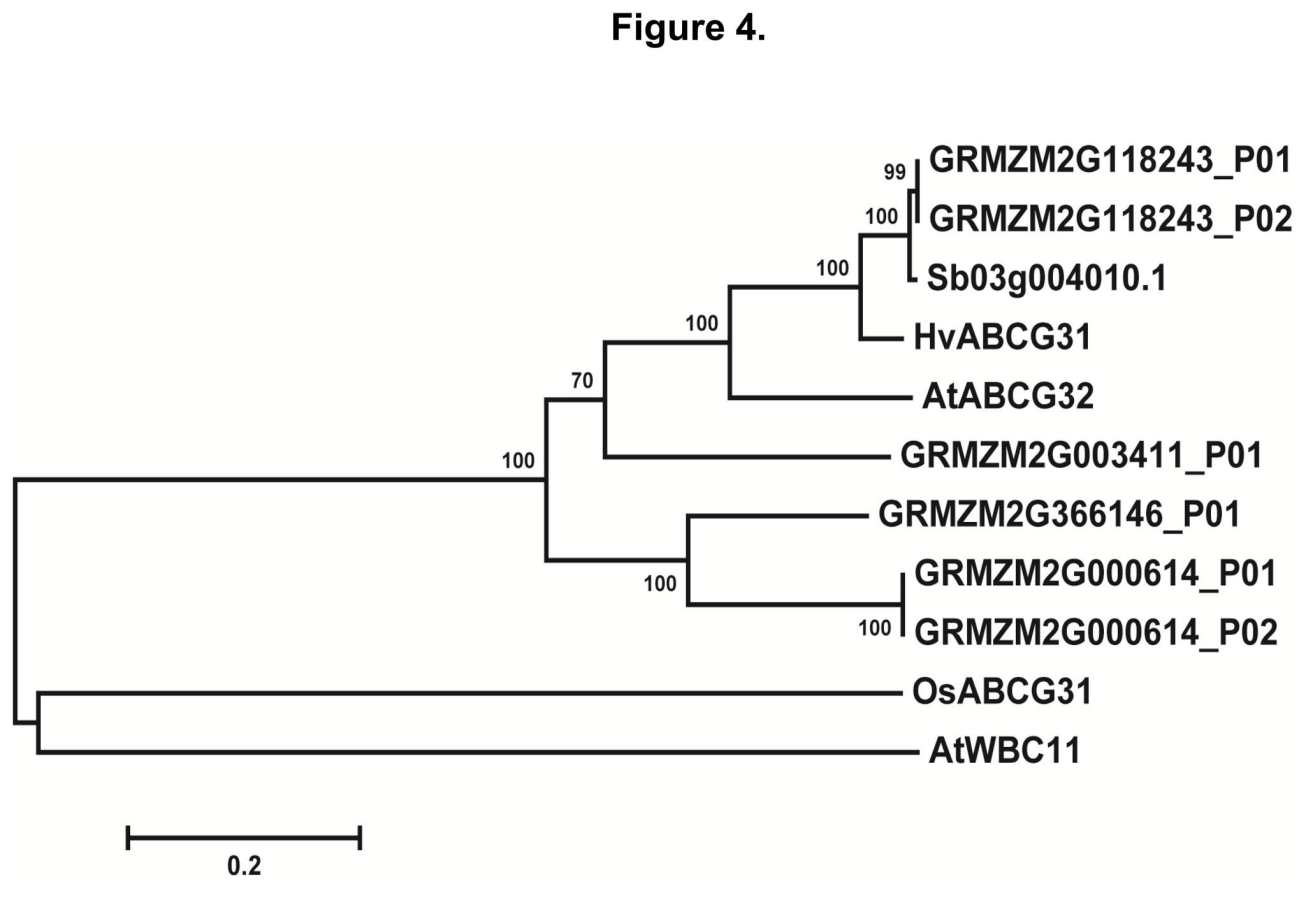
The *gl13* gene encodes a putative ABC transporter G family protein. The amino acid sequence of the 2 GL13 isoforms (GRMZM2G118243_P01 and GRMZM2G118243_P02) were aligned to 9 homologs downloaded from GenBank (Table S2). Neighbor-joining analysis was used to generate a phylogenetic tree, which was bootstrapped over 1,000 cycles, using MEGA5.0.

The amino acid sequence of *gl13* was compared with orthologs from other species in the NCBI database using Blastp. The GL13 protein (encoded by GRMZM2G118243) exhibits 97% similarity to the protein encoded by Sb03g0040410.1 (Sorghum), 94% similarity to OsABCG31 (rice), 92% similarity to HvABCG31 (barley) and 70% similarity to AtABCG32 (*Arabidopsis*). A phylogenetic tree was constructed based on the amino acid sequences of these proteins (Table S2, Figure 4). Domain analysis of the protein encoded by GRMZM2G118243 predicted that *gl13* encodes an ATP-binding cassette subfamily G full transporter, a type of transporter that includes both white-brown complex (WBC) half transporters and pleiotropic drug resistance (PDR) full transporters [30]. Consistent with our phenotyping data, AtABCG32 is predicted to encode an ABCG subfamily transporter involved in the export of epicuticular components from epidermal cells (Table S3) [33,34]. The homologs of *gl13* in rice (OsABCG31) and barley (HvABCG31) both contribute to the accumulation of cutin [34,35].

### Expression and regulation of *gl13*

A Q-Teller analysis (http://www.qteller.com) (Figure S2) showed that the *gl13* gene is highly expressed in tassel [36], young leaves [37,38] and seedling shoots [39], while being expressed at lower levels in seedling roots, seedling leaves (V2 stage), pollen and mesophyll cells, as well as bundle sheath cells [36-39]. 

Of the eight cloned *glossy* genes (*gl1, gl2*, *gl3*, *gl4a, gl8a, gl8b* and *gl15*) only *gl2* was differentially expressed between *gl13* mutant and sibling wild-type seedlings in the BSR-Seq experiment using a FDR=0.05. Surprisingly, the *gl2* and *gl13* genes were both significantly up-regulated in the *gl13-ref* (*gl13-N169*) mutant as compared with wild-type (*q-values=0.032* and *0.042*, respectively) (Table S4).

Based on a published eQTL analysis [40], *gl13* is regulated by a *trans* eQTL located in the 853.4-861.4 cM interval of chromosome 3 that contains five genes. None of the 16 other genes that are also regulated by this eQTL interval are known to be associated with the accumulation of epicuticular waxes (data not shown).

### RNA-Seq analysis of the *gl13* mutant

In our BSR-Seq analysis a total of 30,536 genes were expressed (i.e., accumulated an average of at least five uniquely mapped RNA-Seq reads across samples) in wild-type and/or mutant seedlings. These genes were tested for differential expression between the *gl13* mutant and wild-type (Methods). Controlling the false discovery rate (FDR) at 5%, 8.3% (2,522/30,536) of the expressed genes were differential expressed (DEGs), including 1,533 that were up-regulated (Table S5) and 989 that were down-regulated in the mutant as compared with the wild-type (Table S6). Genes involved in cell wall precursor synthesis, cell wall degradation and secondary metabolism of phenylpropanoids (which is involved in lignin biosynthesis), that encode peroxidases, lipid transfer proteins (LTP) or belong to the APETALA2/Ethylene-responsive element binding protein family which is involved in epidermal wax synthesis [41] are enriched (Methods) among the DEGS (Figure S3, Table S7, S8). Interestingly, most of the enriched pathway annotations are from the up-regulated DEGs rather of down-regulated DEGs (Figure S3, Table S8). Among these DEGs, 541 (35%) of the up-regulated and 49 (4.9%) of the down-regulated genes exhibited a log_2_FC>4 in *gl13* mutants (Table S9, S10). The 541 strongly up-regulated genes are enriched in transcription factors (AP2/EREBP, APETALA2/Ethylene-responsive element binding protein family), biotic stress pathway genes, and cell wall degradation pathway genes (e.g., pectate lyases and polygalacturonases) (Figure S3, Table S7), consistent with roles in the biosynthesis of epicuticular waxes or that are induced by biotic stress.

### *gl13* is up-regulated in EMS-induced alleles

**Figure 5:**
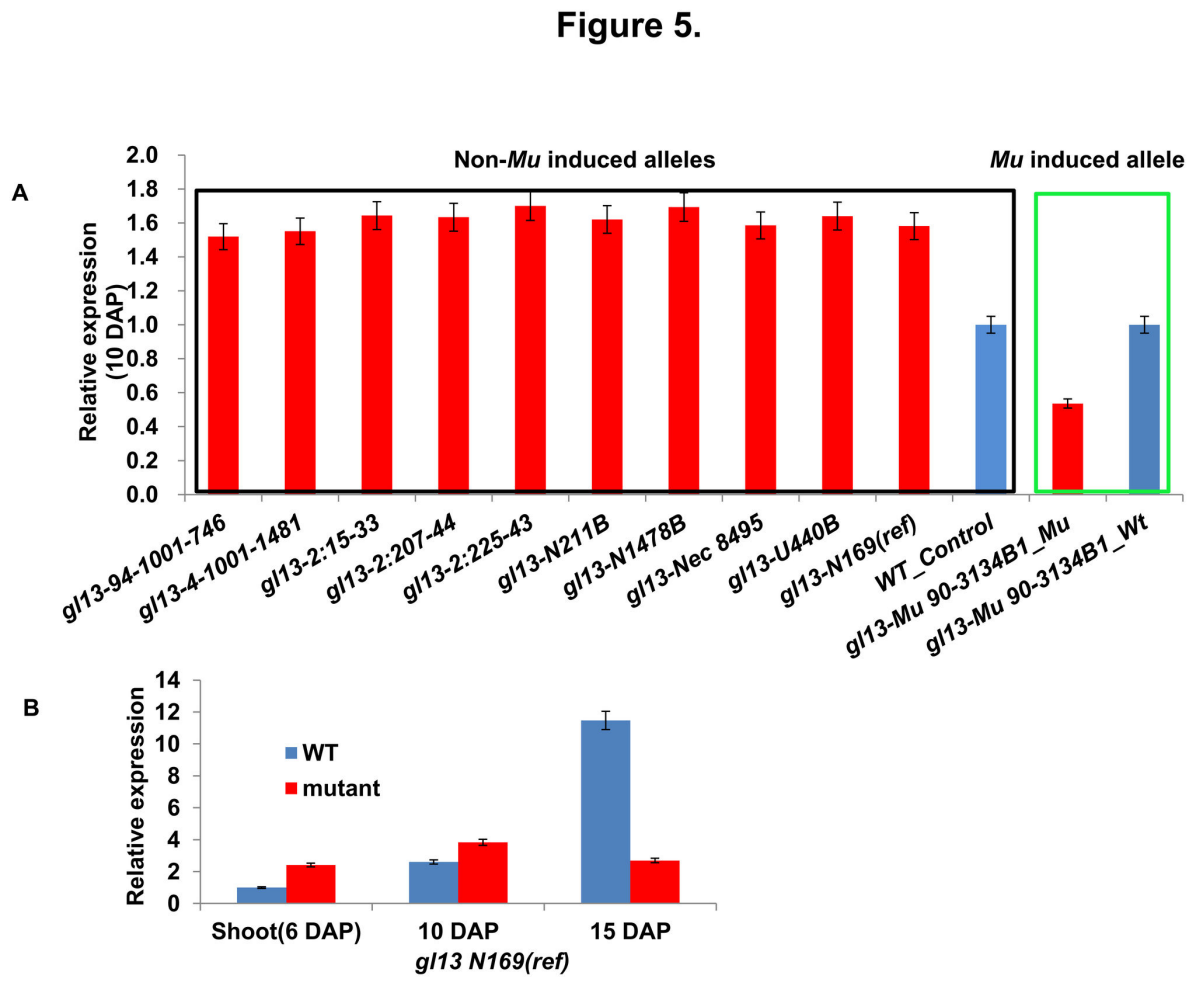
Quantitative RT-PCR analysis of *gl13*. (A) Transcript accumulation from the *gl13* gene was measured via qRT-PCR in 8 EMS-induced alleles and 2 spontaneous alleles and compared to a wild-type control (left box) and the Mu-induced allele and its appropriate wild-type control (right box). (B) The relative expression of *gl13* in shoots (6 DAP) and seedlings (10 DAP and 15 DAP) from wild-type and *gl13-ref* mutant.

Surprisingly, based on the BSR-Seq data, the *gl13* gene was up-regulated in the *gl13* mutant. Consistently, the *gl13* genes was up-regulated in all ten (8 EMS-induced and 2 spontaneous) of the non-*Mu*-induced mutant alleles as compared to wild-type based on RNA-Seq and qRT-PCR analyses of 10 DAP seedlings (Table 3, Figure 5). Only seedlings homozygous for the *Mu*-induced *gl13* allele exhibited decreased expression of *gl13* as compared to wild-type (Figure 5A). In seedlings homozygous for the *gl13-ref* (*gl13-N169*) allele, the *gl13* gene was expressed at higher levels than a wild-type control at both 6 DAP and 10 DAP; in contrast, at 15 DAP, the *gl13* gene was more strongly expressed in wild-type than mutant seedlings (Figure 5B). 

**Table 3:** RNA-Seq expression of 3 EMS-induced *gl13* alleles.

Allele	*gl13-N211B*	*gl13-94-1001-1481*	*gl13-2:207-44*
WT	1.585^a^	2.46	3.474
Mutant	6.506	9.192	9.375

^a^RNA-Sequencing reads information for candidate *gl13*, measured as reads per kilo-base per million reads (RPKM)

## Discussion

During BSR-Seq [17] SNPs are discovered by comparing RNA-Seq reads derived from pools of mutant and wild-type siblings. We used BSR-Seq to map the *gl13* gene to an 8Mb interval of the maize genome. As in any BSR-Seq analysis we were able to define an interval on the genetic map that contains the gene of interest. The SNPs discovered within this mapping interval are available for subsequent experiments, including fine mapping. Moreover, BSR-Seq enables the identification of genes that are differentially expressed between the mutant and wild-type pools. The candidate gene may be one of the differentially expressed genes located within the mapping interval. Differentially expressed genes genome-wide may also provide clues as to the molecular function(s) of the gene of interest. As described above, using the newly developed Seq-Walking technology, we identified a *Mu*-insertion in the coding region of gene GRMZM2G118243 within the *gl13* mapping interval. GRMZM2G118243 was ultimately demonstrated to be *gl13* via sequence analyses of a series of EMS-induced alleles.

Transposons, including the *Mu* transposons of maize [42] are widely used for gene cloning and functional analyses in model organisms [43-48]. After transposon insertion mutants have been isolated, they can be used to clone the underlying genes by identifying the sequences flanking the causative transposon insertions (MFS). Multiple methods have been reported to isolate MFS [49-54]. It is, however, still technically challenging to clone MFS due to the high copy number of *Mu* elements and their continued transposition in “*Mu*-active” lines [55]. In this study, we report a new technology, Seq-Walking to obtain MFS using Life Technologies’ sequencing platform. In combination with the mapping interval defined by the BSR-seq [17] experiment a *gl13* candidate gene was readily defined via Seq-Walking. We began by conducting Seq-Walking on DNA extracted from pools of homozygous mutant and wild-type relatives. This identified large numbers of MFS from each pool, which were then mapped to the B73 reference genome. Only those MFS that mapped to the *gl13* mapping interval defined by the BSR-Seq experiment were used in subsequent analyses. Those MFS that were present in both pools (“universal insertions”) could be discarded. High copy “wild-type specific” MFS present only in the wild-type pool are likely derived from insertions that arose following the divergence of the wild-type and mutant families in this *Mu*-active genetic background. In contrast, “wild-type specific” MFS having low read counts are probably derived from somatic insertions, each of which would be present only in a single seedling in the wild-type pool. We therefore focused on the “mutant-specific” MFS. Because somatic insertions would also be expected to arise in the mutant pools we ranked the mutant-specific MFS by read counts to identify the *gl13* candidate gene. These results demonstrate the ease with which it is possible to map and clone a gene via a combination of BSR-Seq and Seq-Walking if transposon-induced alleles are available. It should be straightforward to adapt Seq-Walking to enable the efficient cloning of sequences flanking other transposons. 

Transposon insertion alleles are, however, not always available. This is the first report of conducting BSR-Seq on EMS-induced alleles. Using EMS alleles in a BSR-Seq experiment has the significant advantage that the mutant alleles would be expected to contain transition mutations (G:C→A:T)[56] in the *gl13* locus. In our search for a *gl13* gene candidate within the 8Mb mapping interval we exploited this fact in parallel with the Seq-Walking experiment described above. Analysis of the three BSR-Seq experiments identified 6, 10 and 5 genes in the *gl*13 mapping interval that harbored non-synonymous typical EMS-induced transition SNPs relative to the B73 reference genome that were also not present in any of the 26 (non-glossy) inbred founders of the NAM population [29]. Nine of the genes in the 8 Mb *gl13* mapping interval were DEG at log_2_FC>=2 (6 down-regulated and 3 up-regulated relative to the wild-type allele; Table S12). We initially made the assumption that the mutant allele of the *gl13* gene would be among the down-regulated genes. Two of the 6 down-regulated genes (GRMZM2G051753 and GRMZM2G352891) were at least potentially involved in wax biosynthesis (Table S9, S10). Neither of these genes, however, contained a typical EMS mutation in any of the three EMS-induced alleles. We next analyzed the 3 up-regulated genes contained within the mapping interval. Two of the EMS-induced mutants contained non-synonymous SNPs that encoded PTC codons in the same gene, GRMZM2G118243, which we subsequently demonstrated was the *gl13* locus. These results demonstrate that by conducting BSR-Seq on EMS-induced mutant alleles it will be possible to at least sometimes directly identify the causative gene from the RNA-Seq data. 

In the case of *gl13* this discovery process was enabled by the fact that all 8 of the EMS-induced and 2 spontaneous alleles accumulated abundant levels of transcripts, indeed these levels exceeded those in the wild-type. This is surprising given that 4/8 of these up-regulated EMS-induced alleles contain PTC mutations (Table 3, Figure 5 red box) and that PTC-containing transcripts are typically degraded via nonsense-mediated decay (NMD) [57]. There are, however, several reports of PTC alleles that are up-regulated as compared to wild-type controls [58-65]. Based on these results we are conducting a global analysis of the expression of PTC alleles among the NAM founders. 

Full-sized ATP-binding cassette (ABC) transporters of the G subfamily (ABCG) are considered to be essential components of the plant immune system [66]. This is consistent with the observation that epicuticular waxes play roles in plant/insect interactions [5]. The fact that excised seedling leaves from *gl13* mutants have lower water retention capacity than leaves from wild-type seedlings indicates that the *gl13* genes, and by extension perhaps, epicuticular waxes in general may play important roles in water relations, as is the case for the *gl13* homologs of barley and rice [34]. These findings suggest a strategy for enhancing drought tolerance in crops. 

## Supporting Information

Figure S1 **Seq-Walking.** Process by which genomic DNA is prepared for Seq-Walking.
(TIF)

Figure S2 **Q-Teller analysis of the *gl13* gene.** Accumulation of transcripts from the *gl13* gene (GRMZM2G118243) in multiple tissues and at multiple stages of development as measured via RNA-Seq.
(TIF)

Figure S3 **Mapman analysis of the results of an RNA-Seq experiment comparing transcript accumulation in wild-type and *gl13* mutant seedlings.** Genes marked in blue and red are up- and down-regulated in mutant relative to wild-type, respectively. Darker shades designate larger fold changes.
(TIF)

Table S1 **Putative causal mutations in 10 *gl13* alleles and the corresponding SNPtypes of the 25 NAM founders based on RNA-Seq reads.** SNP data were discovered from RNA-Seq data of NAM (Nested associated mapping) founders in Schnable lab. The first 8/10 *gl13* alleles shown in column 1 were derived from EMS-mutagenesis. The second and third columns indicate the physical position and nature of the putative causal mutation associated with each allele, if identified. In column 3 the B73 nucleotide is listed first. The SNPs at the putative causal positions are listed in columns 4-30 for each of the 25 NAM founders plus B73 and Mo17). The first number in parenthesis is the number of reads supporting the indicated SNP. The second number is the total reads that align in that particular SNP site. "--" indicates missing data or deletions.
(XLSX)

Table S2 **Homologs of *gl13*s.**
(PDF)

Table S3 **Domain analysis for *gl13* gene.**
(PDF)

Table S4 **Expression of 8 different glossy genes in *gl13* mutant and wild-type seedlings.**
(PDF)

Table S5 **Up-regulated DEGs in *gl13* (mutant vs. wild-type).** The F2 segregating populations of three *gl13* EMS-induced alleles were used for the RNA-Seq experiment (Methods). Maize reference genome version 2 (Refgen2) was used as the reference. Gene orientation and position on chromosomes are listed in the table. RPM represents reads per million of mapped reads. log2FC designates log_2_ of fold changes. *p-value* was from the statistical test of differential expression. *q-value* is an adjusted *p* *value*. sig. indicates whether a gene is significantly differentially expressed.
(XLSX)

Table S6 **Down-regulated DEGs in *gl13* ( mutant vs. wild-type).** The F2 segregating populations of three *gl13* EMS-induced alleles were used for the RNA-Seq experiment (Methods). Maize reference genome version 2 (Refgen2) was used as the reference. Gene orientation and position on chromosomes are listed in the table. RPM represents reads per million of mapped reads. log2FC designates log_2_ of fold changes. *p-value* was from the statistical test of differential expression. *q-value* is an adjusted *p* *value*. sig. indicates whether a gene is significantly differentially expressed.
(XLSX)

Table S7 **DEGs MapMan enrichment analysis.**The enrichment analysis of *gl13* differential expressed genes (DEGs) using MapMan annotation (http://mapman.gabipd.org). Four layers of annotation were defined by MapMan. The enrichment was tested for DEGs, up-regulated DEGs and down-regulated DEGs in each category of each annotation layer. Fisher's Exact test was used for the enrichment test and the resulting *p-values* were adjusted to account for multiple statistical tests (*q-values*).
(XLSX)

Table S8 **Enrichment analysis for DEGs based on Gene Ontology.** Most up-regulated DEGs in *gl13* mutant or wild-type have enrichment information in Gene Ontology database.
(PDF)

Table S9 **541 significantly up-regulated DEGs with at least 16 fold changes in *gl13* mutants relative to wild-type siblings.** Maize reference genome version 2 (Refgen2) was used as reference. Gene orientation and position in chromosome were listed in table. Among 1,533 significantly up-regulated genes in the mutant as compared to the wild-type (Table S5), 541 exhibit at least 16-fold change. (Log_2_FC<=-4).
(XLSX)

Table S10 **49 significantly down-regulated DEGs in *gl13* mutants relative to wild-type siblings.** Maize reference genome version 2 (Refgen2) was used as reference. Gene orientation and position in chromosome were listed in table. Among 989 significantly down-regulated genes in the mutant as compared to the wild-type (Table S6), 49 exhibit at least 16-fold change. (Log_2_FC<=-4). .
(XLSX)

Table S11 **Primers used for *gl13* sequence analysis and Seq-Walking library prepare.**
(PDF)

Table S12 **Differential expressed genes in *gl13* mapping interval.**
(PDF)
