# Correction: The Maize *glossy13* Gene, Cloned via BSR-Seqand Seq-Walking Encodes a Putative ABC Transporter Required for the Normal Accumulation of Epicuticular Waxes

**DOI:** 10.1371/journal.pone.0099563

**Published:** 2014-05-30

**Authors:** The PLOS ONE Staff

There are several errors in the published article.

The legends for Figure 1 and Figure 2 are incorrectly switched. Please refer to Figure legend 2 for Figure 1, and refer to Figure legend 1 for Figure 2.

All instances of *gl13-ref* should appear as *gl13-N169*.

In the Materials and Methods section under Genetic Stocks, the first sentence incorrectly reports the *gl13-N169* accession number as AC520. The correct accession number is AC502. The final sentence of this section mentions allele *gl13-U440B/PI251938b*. For clarification, this should be listed as *gl13-U440B*.

The authors would also like to note that *gl13-Nec 8495* was obtained from E. G. Anderson.
