# Supplementary figures and images for: The Maize glossy13 Gene, Cloned via BSR-Seq and Seq-Walking Encodes a Putative ABC Transporter Required for the Normal Accumulation of Epicuticular Waxes
Source: PLoS One. 2013 Dec 6;8(12):e82333. doi: 10.1371/journal.pone.0082333 (PMC3855708; doi:10.1371/journal.pone.0082333)

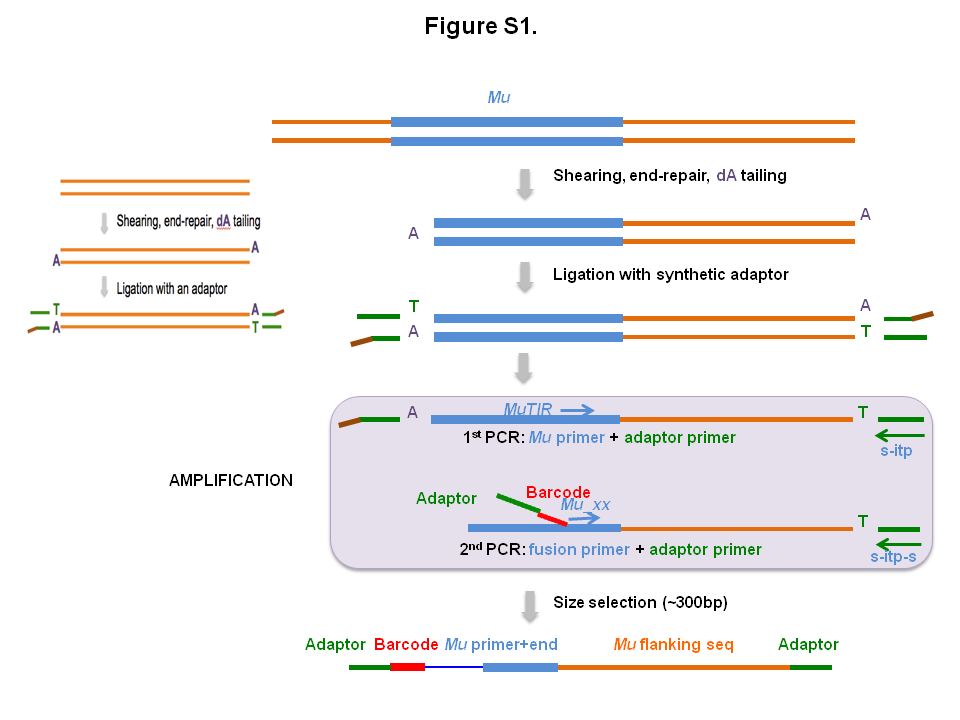

Supplement: Figure S1 — Seq-Walking. Process by which genomic DNA is prepared for Seq-Walking. (TIF) [file pone.0082333.s001.tif]

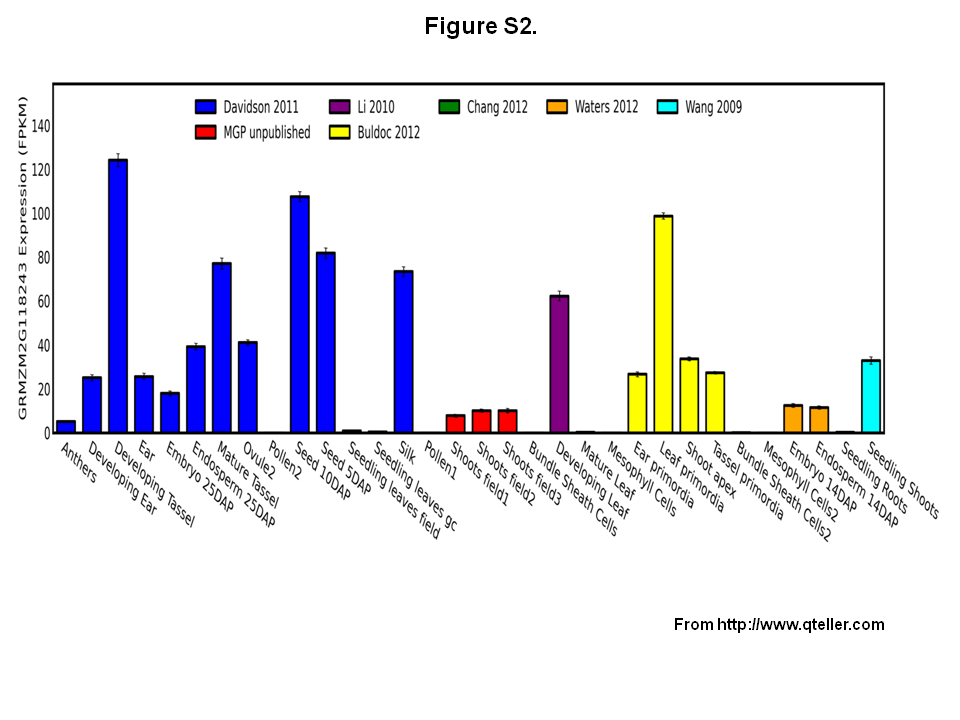

Supplement: Figure S2 — Q-Teller analysis of the gl13 gene. Accumulation of transcripts from the gl13 gene (GRMZM2G118243) in multiple tissues and at multiple stages of development as measured via RNA-Seq. (TIF) [file pone.0082333.s002.tif]

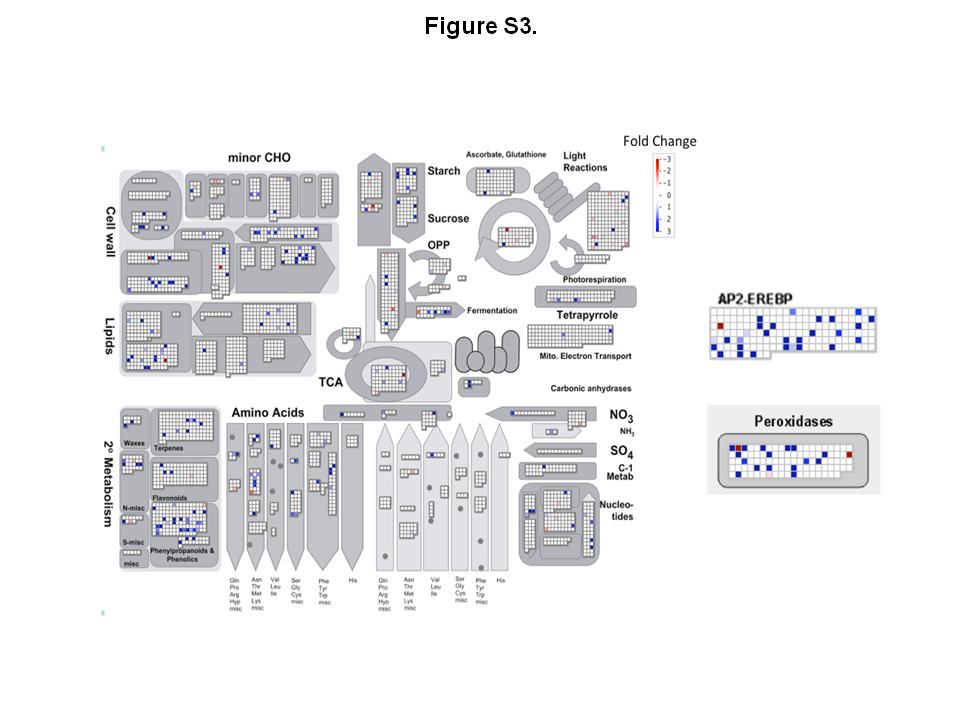

Supplement: Figure S3 — Mapman analysis of the results of an RNA-Seq experiment comparing transcript accumulation in wild-type and gl13 mutant seedlings. Genes marked in blue and red are up- and down-regulated in mutant relative to wild-type, respectively. Darker shades designate larger fold changes. (TIF) [file pone.0082333.s003.tif]
