# Supplementary material for: The Maize glossy13 Gene, Cloned via BSR-Seq and Seq-Walking Encodes a Putative ABC Transporter Required for the Normal Accumulation of Epicuticular Waxes
Source: PLoS One. 2013 Dec 6;8(12):e82333. doi: 10.1371/journal.pone.0082333 (PMC3855708; doi:10.1371/journal.pone.0082333)
Supplement: Table S2 — Homologs of gl13s. (PDF) [file pone.0082333.s005.pdf]

Table S2. Homologs of *gl13* .

Query-ID	Homologs	% identity	*E-value*	bit-score
GRMZM2G118243_P01[a]	**GRMZM2G003411_P01**	56.91 (565/963)	0	1144
	**GRMZM2G366146_P01**	53.46 (376/664)	0	738
	**GRMZM2G000614_P01**	50.61 (531/988)	0	1012
	**AT2G26910.1**	68.44 (637/957)	0	1342
	**Sb03g004010.1**	97.28 (934/957)	0	1884
	**LOC_Os01g08260.1**	91.3 (126/138)	1.00E-71	270
	**HvABCG31**	91.15 (875/960)	0	1778
GRMZM2G118243_P02[a]	**GRMZM2G000614_P01**	53.49 (813/1462)	0	1620
	**GRMZM2G000614_P02**	51.12 (867/1522)	0	1576
	**GRMZM2G003411_P01**	59.51 (867/1440)	0	1768
	**GRMZM2G366146_P01**	55.25 (662/1153)	0	1323
	**AT2G26910.1**	70.45 (1023/1435)	0	2112
	**Sb03g004010.1**	97.76 (1402/1431)	0	2855
	**LOC_Os01g08260.1**	93.95 (575/612)	0	1206
	**HvABCG31**	92.26 (1426/1434)	0	2719

Note: [a], GL13
