# Supplementary material for: The Maize glossy13 Gene, Cloned via BSR-Seq and Seq-Walking Encodes a Putative ABC Transporter Required for the Normal Accumulation of Epicuticular Waxes
Source: PLoS One. 2013 Dec 6;8(12):e82333. doi: 10.1371/journal.pone.0082333 (PMC3855708; doi:10.1371/journal.pone.0082333)
Supplement: Table S3 — Domain analysis for gl13 gene. (PDF) [file pone.0082333.s006.pdf]

Table S3. Domain analysis for *gl13* gene.

Protein ID	Alignment start	Alignment end	Hmm name	Type	Hmm start	Hmm end	Hmm length	Bit score	*E-value*
GRMZM2G118243_P01	183	340	ABC_tran	Domain	1	115	118	3.00E+01	4.70E-07
GRMZM2G118243_P01	497	707	ABC2_membrane	Family	4	209	209	1.39E+02	1.30E-40
GRMZM2G118243_P01	712	775	PDR_assoc	Family	1	64	65	7.99E+01	6.40E-23
GRMZM2G118243_P02	497	707	ABC2_membrane	Family	4	209	209	1.38E+02	2.30E-40
GRMZM2G118243_P02	712	775	PDR_assoc	Family	1	64	65	7.92E+01	1.00E-22
GRMZM2G118243_P02	881	1009	ABC_tran	Domain	1	118	118	3.59E+01	7.00E-09
GRMZM2G118243_P02	1155	1368	ABC2_membrane	Family	2	209	209	1.66E+02	4.30E-49
AT2G26910.1	175	332	ABC_tran	Domain	1	115	118	3.19E+01	1.20E-07
AT2G26910.1	490	699	ABC2_membrane	Family	5	209	209	1.31E+02	2.10E-38
AT2G26910.1	704	769	PDR_assoc	Family	1	65	65	8.53E+01	1.30E-24
AT2G26910.1	870	998	ABC_tran	Domain	1	118	118	3.47E+01	1.70E-08
AT2G26910.1	1144	1357	ABC2_membrane	Family	2	209	209	1.72E+02	7.20E-51

*Hmm: Profile hidden Markov models for protein domain analysis
