# Supplementary material for: The Maize glossy13 Gene, Cloned via BSR-Seq and Seq-Walking Encodes a Putative ABC Transporter Required for the Normal Accumulation of Epicuticular Waxes
Source: PLoS One. 2013 Dec 6;8(12):e82333. doi: 10.1371/journal.pone.0082333 (PMC3855708; doi:10.1371/journal.pone.0082333)
Supplement: Table S4 — Expression of 8 different glossy genes in gl13 mutant and wild-type seedlings. (PDF) [file pone.0082333.s007.pdf]

Table S4. Expression of 8 different glossy genes in *gl13* mutant and wild-type seedlings.

Locus	Gene ID	Encoding protein	Biological Process	Molecular Function	Cellular Component	Expression. In *gl13* (Mu vs. WT)		
						log2FC	*q value*	DEG
gl4a	GRMZM2G003501	Polyketide synthase-like	Fatty acid biosynthetic; lipid biosynthetic process	Catalytic;Acyltransferase; Transferase activity	Membrane	1.46	0.06	no
gl8b	GRMZM2G087323	3-ketoacyl reductase	Fatty acid elongation	Beta ketoacyl-CoA reductase; Reductase activity	Endoplasmic Reticulum	1.05	0.053	no
gl2	GRMZM2G098239	Transferase	-	Transferase activity	-	0.92	0.032	yes
gl1	GRMZM2G114642	Hydroxylase	Fatty acid biosynthetic; Oxidation-reduction process	Iron ion binding; Octadecanal decarbonylase &oxidoreductase activity	Endoplasmic reticulum	0.46	0.104	no
gl13	GRMZM2G118243	Transporter	G-protein coupled receptor; Protein signaling pathway	Nucleotide binding; ATP binding; signal transducer activity; ATPase activity; Nucleoside-triphosphatase activity; Guanyl nucleotide binding	Membrane	1.53	0.042	yes
gl15	GRMZM2G160730	AP2	regulation of transcription, DNA-dependent	DNA binding; sequence-specific DNA binding transcription factor activity	-	-1.69	0.067	no
gl3	GRMZM2G162434	Myb30	-	DNA binding	-	2.48	0.055	no
gl8a	AC205703.4_FG006	Short chain dehydrogenase	Metabolic; Enterobactin biosynthetic; Oxidation-reduction process	Alcohol dehydrogenase (NAD); 2,3-dihydro-2,3-dihydroxybenzoate dehydrogenase; Oxidoreductase& acetoacetyl-CoA reductase activity	-	1.98	0.088	no

Note: FDR 0.05, *P-value* <0.003.
