# Supplementary material for: The Maize glossy13 Gene, Cloned via BSR-Seq and Seq-Walking Encodes a Putative ABC Transporter Required for the Normal Accumulation of Epicuticular Waxes
Source: PLoS One. 2013 Dec 6;8(12):e82333. doi: 10.1371/journal.pone.0082333 (PMC3855708; doi:10.1371/journal.pone.0082333)
Supplement: Table S8 — Enrichment analysis for DEGs based on Gene Ontology. Most up-regulated DEGs in gl13 mutant or wild-type have enrichment information in Gene Ontology database. (PDF) [file pone.0082333.s011.pdf]

Table S8. Enrichment analysis for DEGs based on Gene Ontology

Gene Ontology	Term	DEGs enrich	Mutant enrich
GO:0006869	lipid transport	yes	yes
GO:0008171	O-methyltransferase activity	yes	yes
GO:0016021	integral to membrane	yes	yes
GO:0016491	oxidoreductase activity	yes	yes
GO:0016841	ammonia-lyase activity	yes	yes
GO:0055114	oxidation-reduction process	yes	yes
GO:0005576	extracellular region	no	yes
GO:0005975	carbohydrate metabolic process	no	yes
GO:0006559	L-phenylalanine catabolic process	no	yes
GO:0008289	lipid binding	no	yes
GO:0009058	biosynthetic process	no	yes
GO:0009415	response to water	no	yes
GO:0009607	response to biotic stimulus	no	yes
GO:0015018	Galactosylgalactosylxylosyl protein 3-beta-glucuronosyltransferase activity	no	yes
GO:0016020	membrane	no	yes
GO:0055085	transmembrane transport	no	yes

Note: Most up-regulated DEGs in *gl13* mutant or wild-type have enrichment information in Gene Ontology database (http://geneontology.org/). The DEGs which enriched in the pathway were labeled as yes (column 3) and the mutants enriched genes were shown in column 4.
