# Supplementary material for: The Maize glossy13 Gene, Cloned via BSR-Seq and Seq-Walking Encodes a Putative ABC Transporter Required for the Normal Accumulation of Epicuticular Waxes
Source: PLoS One. 2013 Dec 6;8(12):e82333. doi: 10.1371/journal.pone.0082333 (PMC3855708; doi:10.1371/journal.pone.0082333)
Supplement: Table S11 — Primers used for gl13 sequence analysis and Seq-Walking library prepare. (PDF) [file pone.0082333.s014.pdf]

Table S11. Primers used for *gl13* sequence analysis and Seq-Walking library prepare

oligo_name	oligo_seq(5'-3')	Primer name	Sequence(5'-3')
L_end	GCGATGAGAGAGCAAGTGGT	gl13C1R7	CCAGTCTTCCTGCCAGCTAA
R_end	AGTATCACGCAGGACGAAGG	gl13C1L9	GGATGTTTTAGCTGGCAGGA
L_start	GCCAGTACGCCCTCCTTAAA	gl13C1R9	AGCATACTTCGTGGCGAAAG
R_start	GTATGCGTCGGAAGAAGTGC	gl13C1L11[a]	GGAGCAGATTCTTGGAGTGG
gl13C1L1	GGCAGCTAGGGTTTGGTGT	gl13C1R11[a,b]	CCGAACTGAGAGGTCAGGAG
gl13C1R1.1	ATGATGACGAGGGAGCAAAT	gl13C1L13	GCTCCTGACCTCTCAGTTCG
gl13C1R1.2	TGCCTCAGAAAAGCCTCTGT	gl13C1R13	CACTCTCGCTCCCGTGAC
gl13C1L2.1	CTCCCTCGTCATCATGCTTT	gl13C1L13.2[b]	ACCATTGCGCCTATTATTGC
gl13C1R2.1	GAAGGACGGAACCGTACAAA	gl13C1L14	TCTCGGTGCCCTTTACTTTG
gl13C1L2.2	GTTCCCCAAGATCGAAGTGA	gl13C1R14	GCGAATGATCCAAAGGTGTT
gl13C1R2	ATCCCCGCATTCTTTTCTCT	gl13C1L15	GCAATTCCAGGAGTGCCTAA
gl13C1L3	AAGAATGCGGGGATAAAACC	gl13C1R16	AGGCAACGCTGAATACATCC
gl13C1R3	ACCTGAATCCCATAGCACCA	gl13C1L17	CAAGACTGGCATGCTTCAGA
gl13C1L4	AAGCAGACAAGCCTCGTAGC	gl13C1R17	AGCACAGACGTCCAGTCCTAA
gl13C1R4	GGCACCAATACTGCTGTTGA	gl13C1L18	TCAAGGAACAGAAGGGGATG
gl13C1L5	TGGTGCTATGGGATTCAGGT	gl13C1R18	CCCTTTGAGTGTCTGCATCA
gl13C1R5	CAAAGTAAAGGGCACCGAGA	gl13C1L19	CTGGCATGCTTCAGAAATGA
gl13C1L6	AACTACCAGTGGCAGCGTCT	gl13C1R19	TTGTCAGGCGCTTCTTTTG
gl13C1R6	AACCAAACAATGCTCCAACA	Actin408F[b]	CCAGGCTGTTCTTTCGTTGT
gl13C1L7	TTGGCTCTTTTCCGAGTCAT	Actin520R[b]	GCAGTCTCCAGCTCCTGTTC
s-itp(23mer)[c]	C*CTCTCTATGGGCAGTCGGTGAT		
s-itp-s(20mer)[c]	C* CTCTCTATGGGCAGTCGGT		
Itp_adp_1 [c]	TCACCGACTGCCCttt		
Itp_adp_2/5phos/[c]	TCACCGACTGCCCttt		
MuTIR(31bp)[c]	AGAGAAGCCAACGCCA(AT)CGCCTC(CT)ATTTCGTC		
itp_mu43	5'CCATCTCATCCCTGCGTGTCTCCGACTCAGCGTCTACGCCTCYATTTCGTCGAATC 3'		
itp_mu10C[c]	5'CCATCTCATCCCTGCGTGTCTCCGACTCAGTGCGCTATCGCCTCYATTTCGTCGAATCC 3'		

Note: **a,** primers used for *Mu*-induced *Mu* insertion detect; **b,** primers used for qRT-PCR, Actin primers as reference; **c,** primers used for Seq-Walking library preparation
