# Supplementary material for: The Maize glossy13 Gene, Cloned via BSR-Seq and Seq-Walking Encodes a Putative ABC Transporter Required for the Normal Accumulation of Epicuticular Waxes
Source: PLoS One. 2013 Dec 6;8(12):e82333. doi: 10.1371/journal.pone.0082333 (PMC3855708; doi:10.1371/journal.pone.0082333)
Supplement: Table S12 — Differential expressed genes in gl13 mapping interval. (PDF) [file pone.0082333.s015.pdf]

Table S12. Differential expressed genes in *gl13* mapping interval.

Gene ID	Chr	Start (bp)	End (bp)	*gl13* WT (RPKM)	*gl13* Mutant (RPKM)	*gl13*Mutant. *gl13*WT (log2FC)
GRMZM2G083374	3	9,768,559	9,803,414	51.75	39.35	-0.64
GRMZM2G118243[a]	3	10,273,100	10,283,228	2.51	8.36	1.53*
GRMZM2G146644	3	7,599,914	7,604,652	0.09	1.93	4.12*
GRMZM2G150928	3	12,559,391	12,563,061	2.11	5.35	1.12
GRMZM2G158228	3	9,034,928	9,039,723	110.13	72.2	-0.85
GRMZM2G314898	3	8,299,415	8,303,114	9.38	44.47	2.01*
GRMZM2G319169	3	12,790,693	12,812,174	20.58	13.21	-0.88
GRMZM2G397755	3	5,776,179	5,778,416	0.04	0	-15.99*
GRMZM2G540322	3	11,871,649	11,872,382	1.46	0.01	-7.2*

Note: [a], gl13 gene; * stand for significant differential expressed genes compared with wild-type control (FDR =5%, *q-value* < 0.05).
